# Robotic Sorting and Handling in Food Manufacturing: Enabling Technologies, System Integration, and Industrial Challenges

**DOI:** 10.3390/foods15213991

**Published:** 2026-11-06

**Authors:** Haoyang Xu, Xiaowei Huang, Zhihua Li, Xiangyu Hu, Jiyong Shi, Xiaobo Zou

**Affiliations:** Agricultural Product Processing and Storage Lab, School of Food Science and Engineering, Jiangsu University, Zhenjiang 212013, China

**Keywords:** food robotics, task-level reliability, soft grippers, machine vision, intralogistics, hygienic design

## Abstract

Reliable robotic sorting and handling in food manufacturing remain challenging because foods vary in geometry, mechanical properties, surface condition, quality attributes, and spatial arrangement. These variations create coupled demands on perception, grasping, motion, control, and hygiene that component-level optimisation alone cannot resolve. This review examines grippers, perception and optical sensing, robotic platforms, and control strategies, together with their integration into food handling, sorting, packaging, and intralogistics. A recurring limitation is the gap between component performance and complete task success. Accurate recognition may not ensure a feasible grasp, while greater compliance can compromise force transmission or dynamic stability. Richer sensing can increase latency and integration burdens, and faster robot motion does not necessarily improve useful throughput. Based on the reviewed evidence, this review proposes an evidence-informed uncertainty-matched system-design perspective that links sensing, mechanics, control, and intelligence to product and process variability, dominant failure modes, and operating requirements. Industrial deployment requires validation across representative products, repeated operation, sanitation, failure recovery, and changeovers. Such task-level validation should assess complete systems for product integrity, hygienic compatibility, useful throughput, recoverability, and sustained reliability under realistic manufacturing conditions.

## 1. Introduction

Growing demands for production efficiency, consistent product quality, food safety, and manufacturing flexibility are accelerating automation across the food industry, particularly in sorting and handling, which remain labour-intensive in primary processing, where labour shortages, rising costs, and increasing requirements for precision and hygienic operation further reinforce the need for automated solutions [1]. Industrial robots are widely used in structured operations such as packaging and palletising, yet direct food handling and sorting remain more difficult because foods vary substantially in geometry, mechanical properties, surface condition, and susceptibility to damage [2]. In 2024, 542,076 industrial robots were installed worldwide, including 20,792 in the food sector [3]. Robotic sorting and handling integrate perception, manipulation, and motion control to recognise, grasp, transfer, classify, and place products. Unlike standardised manufactured parts, foods within the same category can differ in size, shape, stiffness, deformability, surface friction, maturity, orientation, and internal quality, affecting both perception and physical interaction.

Advances in machine vision, compliant and soft gripping, three-dimensional sensing, machine learning, and adaptive control have expanded robotic manipulation to more fragile, irregular, and mechanically heterogeneous foods [2,4,5]. Much of this progress is still evaluated at the component level. Researchers assess perception systems by detection or classification accuracy, grippers by grasp success or force regulation, and robotic platforms by speed, payload, or positioning performance. Strong component performance does not necessarily translate into reliable task completion. Accurate detection may still yield an infeasible grasp because of deformation, slipperiness, or unsuitable contact geometry; greater compliance can reduce local stress but limit force transmission or dynamic response; additional sensing can improve observability but requires additional calibration and computation and may increase latency. Reliable food manipulation depends on how these capabilities operate together under changing product and production conditions. Hygienic compatibility adds a further system constraint [6,7]. Components near exposed foods may encounter residues, moisture, detergents, and repeated sanitation, and gripper materials, sensors, joints, seals, and protective structures must remain functional without compromising cleanability or food-contact suitability. Mechanical adaptability and sensing cannot be separated from these requirements because structures that improve gripping or feedback may also increase sealing, cleaning, and maintenance demands.

Previous reviews have addressed food-handling robots, end effectors, robot selection, machine vision, and broader food-manufacturing automation from complementary perspectives [1,8,9,10]. Less attention has focused on how these technologies interact under the combined constraints of product variability, task reliability, cycle time, and hygiene. Improved recognition cannot compensate for unsuitable contact mechanics, and mechanical adaptability cannot resolve inadequate information on product quality, position, or motion. The appropriate level of technological complexity therefore depends on the dominant failure modes and operating requirements of the target process. Mechanically uniform packaged products following predictable trajectories may be handled with relatively simple sensing and conventional robotic platforms [2], whereas fragile, deformable, slippery, randomly arranged, or frequently changing foods can require compliant mechanics, richer perception, closed-loop force regulation, and more adaptive control [11,12,13]. The key issue is therefore not the amount of robotic capability incorporated into a system, but whether the selected capabilities address the variability and failure modes that determine reliable task performance.

**Figure 1:**
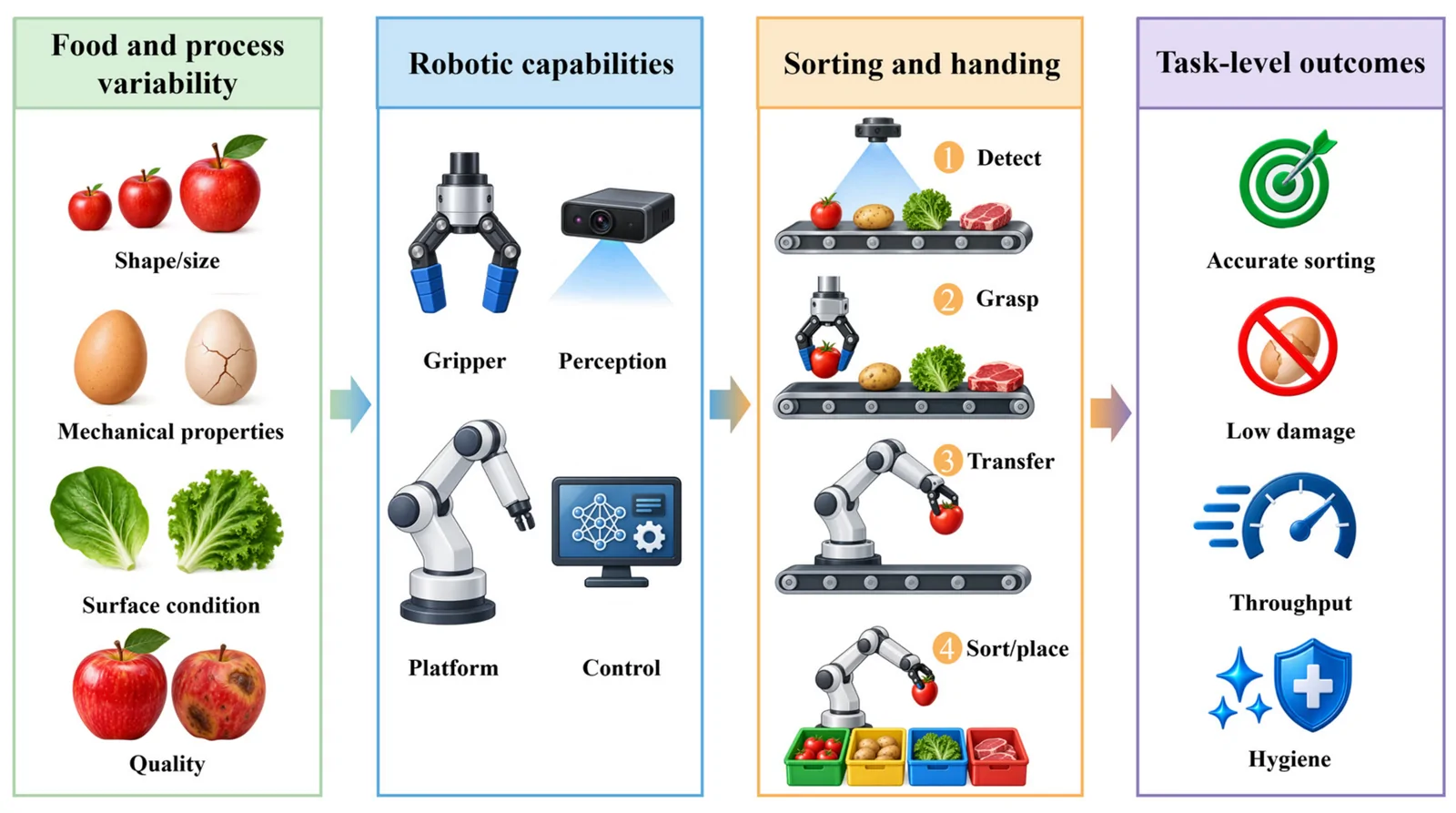
System-oriented framework for robotic sorting and handling in food manufacturing.

From a system-oriented perspective, robotic sorting and handling are considered in terms of the capabilities required for different forms of product and process variability, the conditions under which component-level performance fails to translate into reliable task completion, and the matching of mechanics, sensing, control, and intelligence to dominant failure modes and production constraints. Task-level reliability provides the link between enabling technologies and industrial deployment. An uncertainty-matched system design perspective is adopted as a practical design logic rather than a universal theoretical framework: additional capability is justified when it addresses a task-relevant limitation without creating greater burdens in latency, hygiene, maintenance, integration, or cost. This perspective helps clarify how advances in robotic technologies can be translated into systems that remain reliable, hygienic, and practically deployable under realistic food-manufacturing conditions. Figure 1 summarises the relationship among food and process variability, robotic capabilities, sorting-and-handling tasks, and task-level manufacturing outcomes.

## 2. Literature Search and Evidence Selection

This narrative review was developed through a topic-guided synthesis, supplemented by a literature search in the Web of Science Core Collection conducted on 17 October 2026. The search covered publications from 2015 onwards and addressed robotic food handling, grippers, perception and sorting, system integration and reliability, mobile intralogistics, and related agricultural and postharvest applications. Earlier foundational studies were retained where relevant.

Literature was selected according to its relevance to food-manufacturing tasks, with emphasis on reported system configurations, experimental performance, and operational limitations. Representative studies were selected to cover different robotic capabilities and application settings, taking into account the information available on system design, test conditions, task-related outcomes, and limitations. Studies with limited relevance to the review or insufficient information to assess the task or system performance were not selected as representative examples. Studies focusing solely on food-quality sensing were considered as evidence of sensing capabilities rather than complete robotic performance. For integrated robotic studies, application contexts were classified as direct food-manufacturing (D), adjacent agricultural/postharvest (A), or transferable general-robotics evidence (T), with validation limitations considered separately. The detailed search expressions are provided in Supplementary Table S1.

## 3. Enabling Technologies for Robotic Sorting and Handling

Robotic sorting relies on coordinated end effectors, perception, robotic platforms, and control. The gripper establishes physical contact, perception provides product and spatial information, the platform determines reachable motion and cycle capability, and control couples sensing with manipulation. Their suitability depends on food properties, production speed, workspace, and hygienic requirements [2].

### 3.1. Grippers

End-effector design remains a major challenge in robotic food handling because the contact interface must accommodate variation in geometry, stiffness, deformability, fragility, and surface condition [14,15,16]. Wet, adhesive, or slippery surfaces reduce grasp stability, and irregular shapes or close product spacing complicate contact and separation [11]. Effective grippers must conform to product geometry without imposing excessive local stress and still provide adequate force transmission, response speed, durability, and cleanability. Current strategies address these requirements through mechanical compliance, tunable stiffness, distributed contact, and contact-state sensing.

Soft pneumatic and underactuated grippers provide mechanical adaptation to uncertain product geometry. Pneumatic structures deform through pressurised chambers, artificial muscles, bellows, or related elastomeric elements. The PneuNet architecture introduced by Ilievski et al. [17], for example, generates bending through interconnected channels embedded in compliant materials, allowing the gripper to accommodate shape and positioning uncertainty mechanically. Underactuated mechanisms achieve a similar effect using fewer actuators than mechanical degrees of freedom, with springs, compliant joints, linkages, or soft actuators enabling passive conformity after contact. The RBO Hand 2 demonstrated how structural compliance and underactuation can support multiple grasp configurations with relatively low control complexity [18]. Both approaches reduce dependence on precise geometric matching and are attractive for fragile or variable foods, but increased compliance can limit force transmission, load support, and high-speed response [14,15,19].

Active-material and variable-stiffness grippers extend this trade-off between conformity and force capacity. Shape-memory alloys can embed compact actuation within deformable structures, with recent designs improving heat dissipation or incorporating contact sensing [20,21]. Dielectric elastomer actuators enable electrically driven deformation and substantial bending, although their force output may remain modest, and high driving voltages are often required [22]. SMA actuation remains limited by thermally governed response and recovery, whereas DEA systems require protection against dielectric failure, fatigue, and environmental exposure [20,23]. Variable-stiffness mechanisms instead remain compliant during initial contact and increase rigidity after enclosure or positioning. Magnetorheological materials, particle jamming, and related stiffness-switching mechanisms can improve load support without abandoning geometric adaptability [24,25,26]. Their practical value depends on whether gains in grasp stability justify additional actuation, sealing, control, and sanitation requirements.

Distributed-contact and bioinspired grippers reduce local loading by spreading grasp forces over larger areas [12,27]. Octopus-inspired systems combine compliant structures with suction, whereas tendril-inspired designs use wrapping or entanglement to create redundant contacts. Wang et al. demonstrated a hydrogel gripper with flexible tentacles and switchable bionic suckers for nondestructive manipulation of ultra-soft foods, including tofu and raw egg yolk [28]. Li et al. used multiple compliant tendril-like elements to improve grasp security without precise contact placement [29]. These approaches can improve stability and reduce local pressure, but effective manipulation also requires rapid and predictable release. Folds, cavities, suction interfaces, and multiple contact surfaces may further increase cleaning and hygienic-design demands [6]. Their value therefore lies in the balance among contact stress, grasp stability, release performance, and hygienic accessibility rather than biomimicry itself.

Mechanical adaptation cannot fully compensate for changes in friction, stiffness, deformation, or post-contact motion. Force, tactile, bending, and torque sensing provide information on contact state, slip, and load distribution that external vision may not capture reliably. Liu et al. integrated force and bending sensing into a compliant bionic gripper and used tactile feedback to detect slip and regulate grasping force during fragile fruit and vegetable handling [11]. Such feedback allows the end effector to adapt after contact instead of relying on a fixed, predefined force.

**Figure 2:**
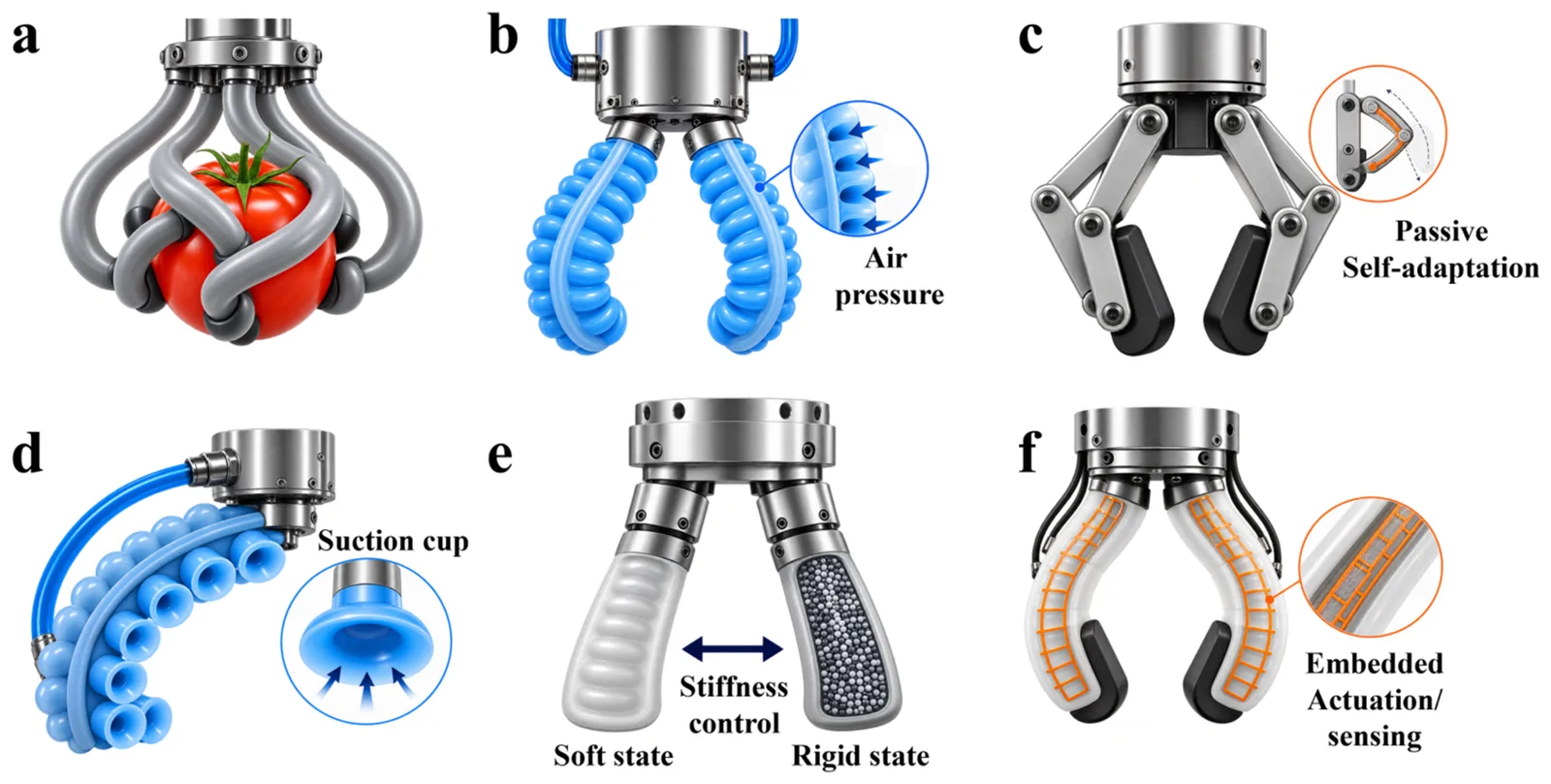
Representative end-effector mechanisms for robotic food handling. Representative end-effector designs include (**a**) tendril- or entanglement-based grippers, (**b**) pneumatic compliant grippers, (**c**) underactuated grippers, (**d**) suction- or octopus-inspired grippers, (**e**) variable-stiffness grippers, and (**f**) active-material grippers.

Sensor integration, however, also affects hygienic design and system complexity. End effectors may contribute to cross-contamination during repeated food handling, and embedded sensors, wiring, joints, and protective structures can increase sealing, calibration, maintenance, and cleaning requirements [6,30]. Additional sensing is therefore most valuable when it resolves contact-state uncertainty that materially affects grasp stability or product protection. No single gripper architecture is suitable for all food products. Gripper selection must balance contact adaptability and load support against release performance, cycle time, and hygienic requirements. Representative end-effector mechanisms are illustrated in Figure 2, and their demonstrated performance and principal capability trade-offs are compared in Table 1.

### 3.2. Perception and Sensing Technologies

Perception converts product variability into information that supports robotic decisions. Depending on the task, a sorting or handling system may need to determine product quality, location, orientation, geometry, or motion before generating a reliable grasp, rejection, routing, or placement action [2]. Different sensing technologies and analytical models provide different parts of this information [34,35], and their value depends on whether the resulting data support timely and reliable downstream decisions [2,36]. Sensor selection should therefore reflect the information required by the task rather than the nominal richness of the acquired data.

Spectral imaging is useful when sorting criteria cannot be determined reliably from surface colour or shape. Multispectral imaging acquires selected wavelength bands, and hyperspectral imaging records denser spectral information with spatial resolution. These techniques can reveal differences associated with chemical composition [37], bruising, disease, contamination, and other quality attributes that remain indistinguishable in conventional RGB images [38,39,40,41]. In robotic sorting, their main role is to convert hidden product attributes into grading, rejection, or routing decisions within the time available for subsequent action. Conventional hyperspectral systems can generate large data volumes and require correction, preprocessing, and inference before a decision becomes available [39,41]. Bian et al. reported an on-chip broadband visible–near-infrared hyperspectral sensor covering 400–1700 nm with 96 spectral channels and 1024 × 1024-pixel imaging at 124 fps, with demonstrations including chlorophyll and sugar quantification and apple bruise detection [42]. Such developments improve the feasibility of online spectral inspection, although illumination sensitivity, calibration stability, computation, and system cost remain important integration constraints.

Point spectroscopy and related optical sensing methods can provide targeted measurements when spatially resolved information is unnecessary [43]. Near-infrared and Raman spectroscopy have been applied to chemical and safety analysis [44,45,46], freshness evaluation [47,48], and chemometric assessments of quality, authenticity, and source attribution [47,49,50]. Their relevance to robotic sorting is strongest when limited measurements can provide an intrinsic-quality decision that cannot be obtained reliably from external appearance. Guo et al., for example, showed that complementary NIR and Raman information improved prediction of salmon freshness based on TVB-N [51]. This provides analytical evidence for generating an intrinsic-quality decision variable rather than validation of a complete robotic sorting system. Deployment still depends on measurement geometry, calibration transfer, matrix variability, and analysis time [52].

Robotic manipulation also requires three-dimensional information about the relationship between the product and the robot. Structured light, time-of-flight imaging, stereo vision, and RGB-D sensing can generate depth maps or point clouds for estimating size, shape, surface geometry, orientation, and position [36]. This information is particularly important for randomly arranged, overlapping, or piled foods, where two-dimensional detection may identify a target without providing a reliable grasp point or approach direction. Yu et al. used binocular structured-light reconstruction to quantify apple deformity, diameter, volume, and mass [53]. Ali et al. developed an active dual-line laser system for high-resolution depth imaging of piled agricultural commodities [54]. These studies show how detailed geometry can support grading, localisation, and object separation in complex arrangements. Greater geometric detail increases acquisition and reconstruction demands, so depth resolution must be balanced against field of view, surface reflectance, processing time, and the latency tolerated by the manipulation task.

Visual sensing data are converted into task-relevant information, such as product category, defect regions, object boundaries, and target position or motion, through classification, object detection, segmentation, and tracking [36,55]. Xu and Lu demonstrated this integration in automated sweetpotato grading on a roller conveyor [55]. Their YOLOv8-based framework combined instance segmentation, tracking, defect evaluation, and grading, with BoT-SORT supporting multi-view tracking. The system achieved 91.7% overall grading accuracy across four quality classes. Its significance lies in integrating several perceptual functions within continuous product flow rather than optimising a single detector. Transfer to different batches and production environments still depends on illumination, product appearance, dataset diversity, and inference speed.

Dynamic production conditions further restrict the time available for perception. High-speed vision combines rapid acquisition with low-latency detection and tracking so that product information remains usable during conveyor motion. Kurniawan et al. developed a real-time foreign-material inspection system for fresh-cut cabbage and green onion using a CMOS camera, YOLOv7x detection, and conveyor control [56]. Detection performance declined when conveyor speed increased from 36.73 to 69.88 cm s^−1^ because of motion blur, partial occlusion, and incomplete feature capture. This finding shows that perception performance is coupled to production conditions rather than being an isolated property of the detector. Models that perform well on static or offline data may provide weaker task performance after line integration [57]. Faster product motion shortens acquisition time, and computationally intensive models can increase inference delay. Image-level accuracy should therefore be considered together with latency and the mechanical response time available for grasping, rejection, or placement.

Multimodal sensing is useful when no single source provides all the information required for action. RGB images describe surface appearance, depth sensing provides geometry, spectral and aroma-related analytical measurements reveal quality attributes [58,59,60,61], and tactile or force sensing can confirm contact conditions after grasping [11]. X. Chen et al. combined RGB images with LiDAR point-cloud information for robotic grasping of thin-skinned fruits, linking classification and instance segmentation with spatial information for grasp planning [62]. The value of multimodal sensing lies in resolving complementary information gaps rather than increasing data volume for its own sake. Additional modalities introduce registration, synchronisation, calibration, processing, maintenance, and hygienic-protection requirements, especially in environments exposed to food residues, moisture, and repeated cleaning [6]. A multimodal architecture is most useful when the additional information resolves a limitation that cannot be addressed adequately by a simpler configuration.

**Figure 3:**
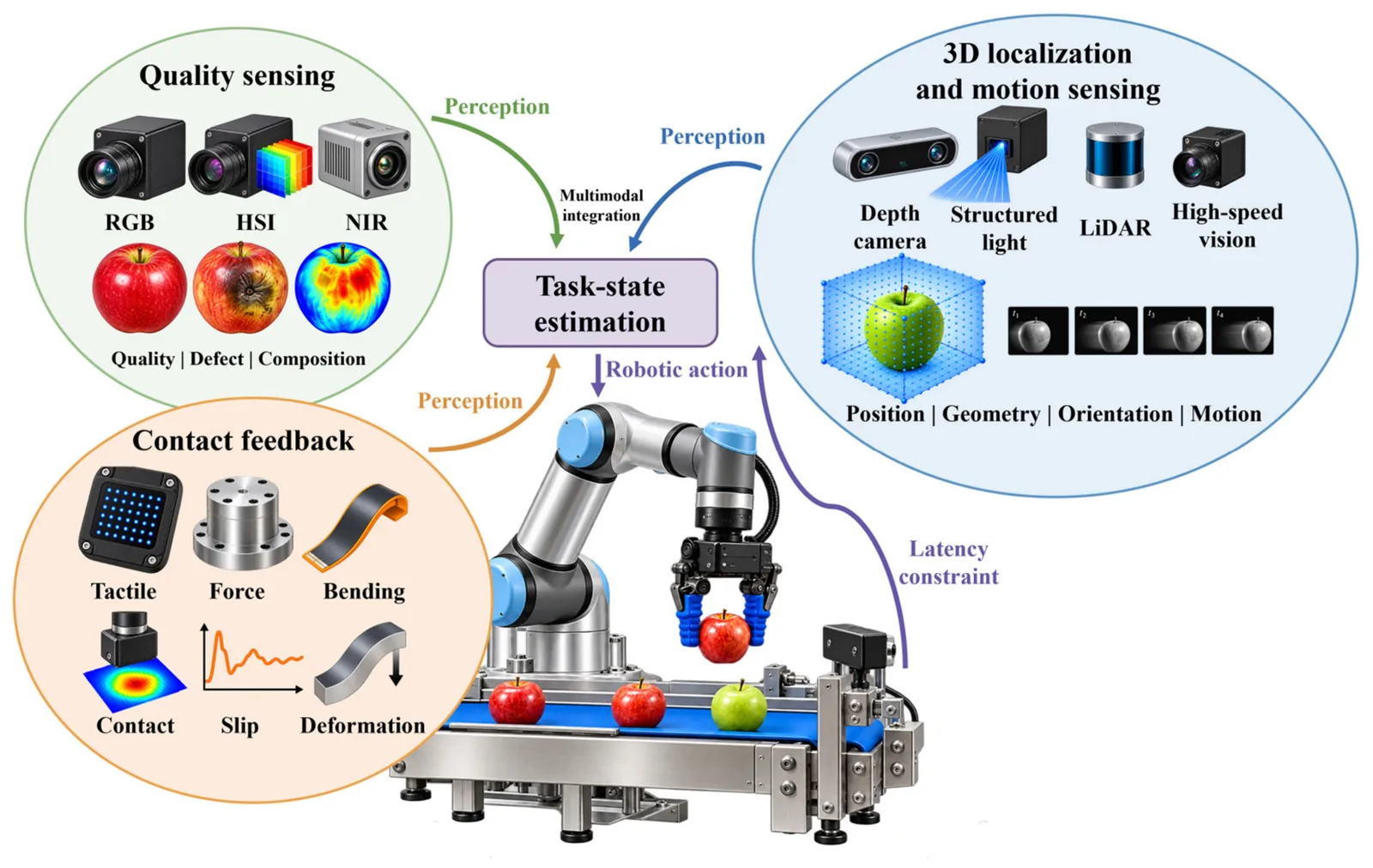
Task-oriented sensing architecture for robotic food sorting and handling.

For manufacturing integration, sensor selection also depends on when information is needed within the task cycle [63]. Food-safety screening [64,65,66,67] and origin verification [68] can inform upstream inspection decisions, while depth and high-speed vision support localisation and interception, and tactile or force sensing provides feedback after contact. This stage-specific allocation can reduce unnecessary data fusion and associated integration demands. Figure 3 organises the main sensing modalities by the task-relevant states they provide, and Table 2 summarises representative approaches, information outputs, and integration constraints.

### 3.3. Robotic Platforms

Selection of robotic platforms in food manufacturing requires balancing workspace, payload, cycle time, positioning accuracy, dexterity, cleanability, and production-line integration. Fixed-base manipulators include Cartesian, SCARA, articulated, and parallel architectures, each offering a different combination of motion range, speed, and orientation capability [2]. Mobile platforms extend operation beyond a fixed workstation when products, materials, or destinations are distributed across a wider production space. Collaborative operation is better considered a safety and interaction mode than a distinct kinematic architecture [70]. Platform selection should begin with the physical and operational requirements of the task rather than a preferred robot category.

Cartesian and SCARA robots are suited to structured operations with predictable motion and limited orientation changes. Cartesian systems provide simple linear movement for repetitive transfer, loading, and case handling, and SCARA robots offer rapid planar motion within compact workspaces for pick-and-place and packaging. Their relatively simple kinematics support high repeatability and short cycle times; however, their restricted orientation limits tasks requiring complex three-dimensional access. Articulated robots provide greater spatial flexibility through multiple rotary joints and can vary end-effector orientation when product location, access direction, or tool posture changes during handling, cutting, sorting, or packaging [2]. This flexibility increases kinematic and control complexity. Parallel robots, particularly Delta configurations, favour rapid repetitive manipulation of lightweight products. Their low moving mass supports high acceleration and short pick-and-place cycles, making them well suited to conveyor-based sorting and packaging. More complex access, heavier payloads, or large orientation changes favour other architectures.

These fixed-base platforms represent task-dependent compromises rather than a hierarchy of capability. Cartesian and SCARA systems favour simplicity and repeatability, Delta robots favour high cycle rates for lightweight products, and articulated manipulators provide greater workspace and orientation flexibility. Maximum speed, payload, or dexterity is not sufficient as a selection criterion if added capability increases cost and control complexity without addressing the dominant process limitation.

Mobile robotic platforms become relevant when manipulation or transport cannot be confined to a fixed workstation. Autonomous mobile robots and mobile manipulators can connect processing, storage, packaging, and delivery locations as material routes change. Mobility expands the accessible workspace and introduces localisation, obstacle detection, route planning, docking, scheduling, and coordination requirements [71,72]. Mobile manipulation also couples base-positioning accuracy with end-effector positioning, making reliable product acquisition and placement dependent on both navigation and manipulation accuracy [73]. Fixed equipment remains attractive when source and destination locations are stable; mobility becomes more valuable when production areas share robotic resources or transport destinations vary. Its benefit must justify the added traffic-management, docking, scheduling, and material-transfer complexity.

Collaborative operation adds a further design dimension when human and robotic workspaces overlap. Collaborative robots commonly use force or torque monitoring, speed and separation control, and collision-safety functions, supporting tasks in which human judgement or dexterity remains valuable and repetitive or ergonomically demanding work is automated [70,74]. They can be useful in flexible handling, secondary packaging, inspection support, and processes with frequent changeovers. Safety-related limits on speed and force can reduce their suitability for high-throughput repetitive sorting when enclosure or separation from workers is feasible.

Food-manufacturing platforms must also satisfy requirements not captured by payload, reach, and repeatability. Surface accessibility, cleanability, drainage, and compatibility with repeated cleaning can determine whether a technically capable robot is suitable near exposed foods [6]. The required level of protection depends on product exposure: equipment outside the direct food-contact zone may face less stringent demands than platforms exposed to residues, washdown, or cleaning agents. Cleanability should be considered together with workspace and cycle performance.

Platform choice is ultimately a task-matching problem and should be evaluated against the bottleneck of the surrounding production line. Structured repetitive processes favour simpler fixed architectures, lightweight high-speed handling favours parallel systems, variable access favours articulated manipulators, and spatially distributed operations may justify mobility. The selected platform must also remain compatible with the gripper, perception system, control architecture, production layout, and hygienic environment.

### 3.4. Robot Control and Coordination

Robot control links perception, manipulation, and platform motion by converting task requirements into coordinated actions at the joint, end-effector, and system levels. In food sorting and handling, control performance is influenced by robot dynamics, payload variation, product motion, contact conditions, and changes in the surrounding workspace. Joint and trajectory control maintain repeatable motion, force-aware control regulates physical interaction, visual feedback compensates for target displacement, and navigation and coordination methods extend control beyond a fixed workstation. The required level of control complexity depends on the dominant source of variation and whether corrective action can be completed within the available cycle time.

Joint-level motion control provides the basis for accurate and repeatable movement under changing payloads and external disturbances. PID, model-based, sliding-mode, fuzzy, and related approaches have been widely investigated, but their practical relevance to food robotics lies in maintaining stable motion at a useful production rate rather than in controller type alone. Li et al. combined sliding-mode control, fuzzy control, and an improved bat optimisation algorithm for trajectory tracking of a four-degree-of-freedom parallel food-sorting robot, achieving 99.90% sorting accuracy with an average sorting time of 0.509 s [75]. Urrea et al. developed type-1 and interval type-2 fuzzy control strategies for a four-arm Delta manipulator and evaluated trajectory tracking under realistic dynamic conditions. For industrial sorting, disturbance-tolerant motion at the required cycle rate is more informative than maximum tracking accuracy in isolation [76].

Trajectory planning determines how the robot moves between task states and becomes particularly important when products are fragile, moving, or difficult to access. Kinematic and dynamic models can constrain position, velocity, acceleration, and actuator demand while preserving smooth motion. Zhu et al. integrated time-continuous trajectory generation with robust tracking in a dual-arm apple-harvesting robot and validated the complete system under commercial orchard conditions [77]. Liu et al. applied multi-objective trajectory optimisation to a Delta food-sorting robot, jointly considering execution time, energy consumption, and motion stability; the optimised trajectory reduced operating time by more than 9.5% while maintaining sorting accuracy above 97.5% [78]. These results show that trajectory design should balance cycle time, positioning accuracy, smoothness, actuator demand, and product sensitivity rather than optimise speed alone.

Accurate motion does not ensure safe manipulation after contact. Fragile, soft, slippery, or deformable foods require sufficient gripping force for stability without exceeding damage tolerance. Force and compliance control address this problem through impedance, admittance, closed-loop force regulation, and related strategies that adjust physical interaction during grasping. Wang et al. developed a closed-loop force-control system for a pneumatic soft gripper using a matrix force sensor and double-loop architecture, reporting a maximum steady-state force error of 0.07 N under the tested conditions [31]. Y. Liu et al. combined contact-force and bending measurements with slip detection to optimise grasping force during manipulation of fragile fruits and vegetables [11]. Q. Wang et al. used tactile and pneumatic-state information to estimate product-specific adaptive grasping force, further shifting control from fixed setpoints towards regulation matched to the current product and contact state [69]. Contact feedback is most valuable when stiffness, friction, deformation, or slip cannot be predicted reliably before grasping. This benefit must be weighed against calibration, response time, sensor durability, protection, and cleanability requirements during repeated operation and cleaning [6,63].

Product motion creates another control requirement because a target position estimated before robot movement may become inaccurate during conveyor transport, manipulation, or observation from a moving platform. Visual servoing reduces this mismatch by updating robot motion from image features or estimated pose during execution. Park et al. developed a robust six-degree-of-freedom visual-servoing system for cucumber harvesting under target motion and reported 90.0% perception accuracy and 82.22% approach accuracy [79]. The difference illustrates that reliable perception does not automatically yield equivalent manipulation accuracy. M. Chen et al. integrated visual feedback with localisation, locomotion, and dynamic picking to support continuous robotic operation across an orchard rather than evaluating these functions independently [80]. Practical performance depends strongly on latency: image acquisition, inference, coordinate transformation, trajectory updating, and actuator response all occur within the same manipulation window. A visually accurate system may still fail if updates arrive too late to compensate for target motion, and greater image or model complexity can consume the available correction time. Visual servoing should be evaluated as a complete perception–control loop, with perception accuracy, update rate, approach accuracy, and mechanical response considered together.

Navigation extends the control problem beyond a fixed robotic cell. Mobile platforms require localisation, mapping, route generation, local obstacle avoidance, and repeated path updates as workers, equipment, or other robots move through the environment [81]. LiDAR, cameras, and depth sensors can support these functions, but the practical objective in food manufacturing is safe and predictable movement within production constraints rather than algorithmic sophistication alone. Facility-specific hygienic zones, restricted-access areas, processing equipment, and cleaning procedures can further constrain route planning. Navigation should be evaluated together with production layout and material-flow requirements rather than solely through path length or localisation accuracy.

Multi-robot systems add task allocation and shared-resource coordination. Several robots may compete for the same workspace, transport route, workstation, or production resource, so individually optimised motion may not maximise overall system efficiency. Qiu et al. integrated task assignment and path planning for heterogeneous warehouse robots while explicitly considering cooperation among robot types and conflict-free movement [82]. These principles are transferable to food-manufacturing intralogistics, where multiple robots may need to coordinate material supply and removal without interrupting processing or packaging. Similar coordination constraints arise when several fixed manipulators operate around a common conveyor or packaging region, where collision avoidance, product assignment, and downstream capacity can limit the benefit of increasing individual robot speed.

Across these control levels, feedback is most valuable when it addresses variation that materially affects task performance. Joint and trajectory control maintain repeatable motion, force and tactile feedback regulate product contact, visual servoing compensates for target displacement, navigation manages spatial variation beyond the workstation, and multi-robot coordination addresses shared resources and material flow. More sophisticated control is justified when it resolves a failure mode that cannot be handled reliably by a simpler architecture. Its value decreases when sensing latency, actuator response, calibration, computation, communication, or integration becomes the dominant limitation. Control strategy should be selected together with the gripper, perception system, robotic platform, and production constraints. Representative control and coordination approaches across motion, contact interaction, visual feedback, and multi-robot operation are compared in Table 3.

## 4. Robotic Integration Across Food-Manufacturing Tasks

### 4.1. Direct Food Handling

Robotic food handling differs from manipulation of standardised industrial objects because product geometry and mechanical state vary between items and can change during contact. Reliable handling requires more than grasp acquisition: the product must be localised, grasped without excessive deformation, stabilised during motion, transferred, and released within the available cycle time. Application-level evaluation should focus on the complete sequence rather than end-effector performance alone [2,15].

Geometric variability is a common source of handling failure. Compliant and underactuated end effectors can absorb part of the mismatch between expected and actual product shape, reducing dependence on precise object models [16]. Kai et al. used monocular visual feedback to control an underactuated hand for brittle and soft foods without internal markers or dense sensing. Ten of 12 tested foods were handled successfully; two could not be grasped stably without damage [84]. This result illustrates both the value and limitation of passive adaptation: compliance broadens the range of handleable products without guaranteeing stable manipulation across substantially different mechanical properties. More constrained tasks may favour simpler task-specific structures. Endo and Otomo demonstrated controlled food portioning and placement using a two-degree-of-freedom gripper, and recent topping systems have integrated grasping with weighing and dispensing [85,86].

Fragile foods create a narrower operating range because grasp stability must be achieved without exceeding damage tolerance. An et al. developed a linkage-integrated Fin-Ray gripper for adaptive tomato grasping [87], while Zhang et al. demonstrated a soft-containing pneumatic gripper for high-speed handling of breadcrumb-coated oysters [4]. In a related study involving raw oysters, Zhang and Wang examined the effects of pneumatic actuation pressure on grasping force, product stability, and damage during robotic pick-and-place operations. The system completed 15 successful transfers in 15 trials under the selected operating conditions, whereas insufficient or excessive pressure compromised handling stability or product integrity [88]. Together, these studies highlight how food condition, contact geometry, actuation pressure, and robot motion determine the balance between grasp stability and damage prevention. The experiments provide direct evidence for robotic food-handling tasks, although sustained operation under industrial production and sanitation conditions remains to be demonstrated.

Grasp stability can also change after contact, particularly for wet, slippery, adhesive, or deformable products. Y. Liu et al. integrated tactile sensing with a compliant gripper and used slip detection to adjust grasping force during fragile fruit and vegetable handling [11]. Wei et al. combined pressure and slip perception in a super-hydrophobic tactile sensor for real-time grasp-force regulation [89]. These systems show why pre-contact vision may be insufficient once friction, deformation, and load redistribution determine stability. Feedback is most valuable when post-contact conditions remain unpredictable.

Surface interaction affects both grasp acquisition and release. Low friction can require greater normal force and increase bruising risk, and adhesive foods may remain difficult to release after a stable grasp. Hao et al. showed that fingerprint-inspired surface textures improved soft-finger gripping under lubricated conditions [90], and textured compliant pads have also improved frictional stability under wet and dry contact [91]. Connolly described a dedicated end effector for sticky food products, highlighting the need to consider release as well as grasp stability [92]. Food–gripper interaction depends on friction, adhesion, contact area, and release behaviour, not force magnitude alone. Surface modification can improve stability without simply increasing gripping force, provided cleanability remains suitable for food contact.

Hygiene introduces a handling requirement rarely captured by conventional grasp benchmarks, as foodborne contamination risks [93,94] require appropriate control and, where needed, microbiological verification [95,96]. Product residues can alter friction and sensor response, and repeated sanitation can affect compliant materials, seals, and embedded sensing. Pettersson et al. integrated vision, force control, and hygienic design in a gripper that handled a mixed stream of fruits and vegetables without intermediate adjustment; decontamination also reduced cross-contamination by *Listeria innocua* [7]. Chauhan et al. showed that robotic handling can influence both fruit damage and pathogen transfer [30]. Together, these studies link mechanical handling with food-safety evaluation, a combination still uncommon in laboratory demonstrations.

Robot motion can further alter the mechanical conditions established during grasping. Ji et al. compared grasping postures and separation trajectories for apple picking with a flexible three-fingered end effector and found that a circular pull-down pattern with a specific finger orientation produced the lowest grasping forces [97]. The transferable principle is that end-effector design and robot trajectory cannot be optimised independently when damage depends on forces generated throughout manipulation. Compliance provides limited protection if approach or separation motion is poorly matched to the product, especially as acceleration and inertial loading increase during high-speed handling [15].

The distinction between successful grasp mechanics and autonomous handling becomes clearer when components are embedded in a complete task. Roshanianfard and Noguchi combined grasping and stem cutting in a pumpkin-harvesting end effector, but target coordinates were entered manually [98]. Pi et al. demonstrated effective apple grasping without integrating visual positioning into the reported system [99]. These studies establish end-effector capability rather than a complete autonomous perception-to-action sequence. More recent systems integrate vision-derived target information with adaptive soft-gripper control [100]. Reliable operation requires localisation, approach planning, grasp execution, transfer, release, and recovery within the same task cycle.

**Figure 4:**
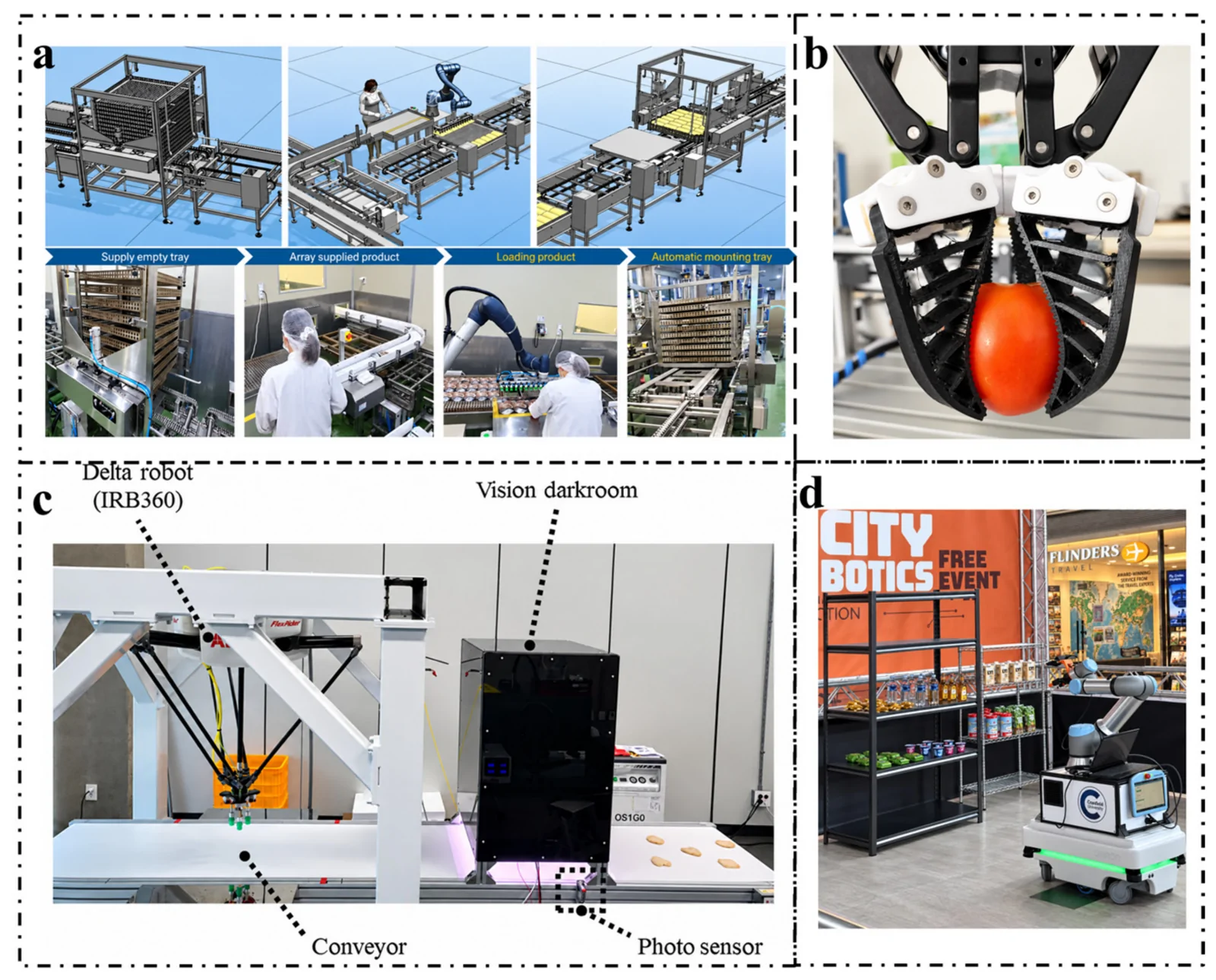
Representative task-matched robotic systems for food sorting and handling. (**a**) Structured and repetitive handling; (**b**) compliant handling of fragile foods; (**c**) high-speed vision-guided conveyor sorting; and (**d**) flexible mobile manipulation. Panels (**a**–**d**) were adapted from [101,102], respectively, under the Creative Commons Attribution 4.0 International License (CC BY 4.0).

Direct food handling should be evaluated at the task level using measures such as grasp success, product damage, transfer stability, placement and release success, cycle time, repeatability, failure recovery, and hygienic compatibility [15,30]. Compliance reduces sensitivity to geometric mismatch, tactile feedback improves post-contact regulation, and suitable trajectories can reduce mechanical loading, but none guarantees complete handling performance in isolation. Reliable operation depends on coordinating contact mechanics, sensing, motion, and sanitation across the expected range of product variation. Representative task-matched robotic systems spanning structured handling, compliant manipulation, conveyor-based sorting, and flexible mobile manipulation are shown in Figure 4.

### 4.2. Sorting and Packaging Operations

Robotic sorting and packaging require perception, grasping, and motion to operate as a coordinated task sequence. Food products and packages may vary in material properties [103,104,105,106], geometry, orientation, surface appearance [107,108], transparency, deformability, and spatial arrangement [2]; some packages also contain functional inserts, including oxygen absorbers [109,110,111]. A target must be detected and localised, physically accessible to the selected end effector, transferred without damage or slip, and placed at the correct destination within the available cycle time. The central challenge is converting perceptual information into executable and repeatable physical action. Performance should be evaluated across this perception-to-action chain rather than through the accuracy of a single module [2,112].

Visual variability is often the first source of failure. Reflective or transparent packaging can create glare and uneven intensity, and random stacking introduces occlusion and ambiguous object boundaries [2,113]. Mojtahedi et al. combined image processing with a Delta robot and a two-fingered gripper for packaging randomly positioned chocolates, achieving an overall pick-and-place success rate of 82% [114]. Strong visual recognition alone does not establish equivalent physical performance. Chakraborty and Deka reported an mAP50 of 94.65% for multimodal litchi detection under occlusion and overlap. However, detection accuracy alone does not establish whether a target can subsequently be separated, grasped, transferred, or packaged. Perception must remain reliable under actual product-presentation conditions, and successful detection represents only the first stage of the complete manipulation process [115].

Grasp estimation narrows this gap by converting visual information into candidate manipulation poses. Cao et al. developed grasp-detection models for 11 fruit categories varying substantially in shape and size and reported detection accuracies of 83.86–93.64% [116]. A predicted grasp remains an estimate of physical feasibility because deformation, surface friction, contact location, allowable force, neighbouring products, and end-effector geometry can alter the interaction after contact [15]. Three-dimensional sensing can reduce part of this mismatch when depth, orientation, or accessibility determines whether a grasp is executable. X. Liu et al. combined Mask R-CNN with structured-light reconstruction for food-package sorting, using image segmentation to identify packages and three-dimensional reconstruction to provide grasp coordinates. The system achieved 100% grasping success for three tested package types [113]. The result demonstrates the benefit of linking semantic recognition with spatial reconstruction, although broader transfer across package materials, geometries, and production conditions remains unresolved.

Information requirements can change after contact. Vision supports localisation and grasp planning, but deformation, slip, and partial occlusion can make the mechanical state less observable once the product has been engaged. Yao et al. combined visual and tactile sensing in a litchi combing-and-cutting end effector, using vision for localisation and tactile signals to assess clamping state. Fruit-stem perception increased from 16.7% with static tactile sensing alone to 86.7% when visual and tactile information were combined [117]. The study provides transferable evidence for stage-specific sensing: external perception guides initial interaction and contact sensing verifies the physical state after grasping. Additional modalities are most useful when the resulting information improves subsequent robotic action enough to justify added calibration and integration requirements.

Complete packaging systems provide stronger evidence of where the perception-to-action chain fails. Yourdkhani et al. [118] developed an automated catering-package assembly system integrating a Delta robot, YOLOv5 detection, FastSAM segmentation, and a two-fingered gripper. Across the class-level trials reported by the authors, the mean physical-grasping success rate was 81.7%. Tea bags were detected in 7 of 10 trials but were not successfully grasped in any of the 10 physical-grasping trials. The authors additionally reported an 85% success rate for autonomous package assembly, although the denominator for this package-level result was not specified. These findings show that reliable detection does not necessarily translate into successful physical manipulation [118].

Zeng et al. examined robotic food assembly using image-based six-dimensional pose estimation and a closed-loop packing strategy for randomly arranged, visually similar food items. The reported success rates were 97.9% for recognition and pose detection and 90% for the closed-loop assembly process [119]. These results extend the discussion from perceptual estimation to the completion of a specified arrangement. Because the reported outcomes were obtained under different experimental protocols, their percentages should not be interpreted as a direct measure of task-stage losses or compared numerically with the catering-package results.

Sorting and packaging can therefore be evaluated as a sequence of distinguishable task stages. Detection determines whether the target is recognised, localisation determines where the robot should act, grasp estimation identifies a candidate interaction, physical grasping establishes contact, transfer tests whether contact remains stable during motion, and placement or assembly determines whether the required final state is reached [112,120]. Failure at one stage limits the practical value of strong upstream performance. Stage-specific evaluation is more informative than a single perception or grasp metric because it identifies where further technical improvement is required.

Robustness can be introduced at both perceptual and mechanical levels. Ummadisingu et al. trained an instance-segmentation model entirely on synthetic food images and transferred it to physical scenes, reducing dependence on manually annotated real data [121]. The same system used spring-loaded fingers that could retract passively by up to 22.5 mm during contact. Synthetic training broadened visual variation represented during learning, and passive finger motion reduced sensitivity to geometric and positioning mismatch during grasping. Simulation still required substantial computational effort for complex food models, showing that synthetic data can reduce some data-collection demands without eliminating development cost.

Task-level benchmarking provides a direct means of identifying whether failure originates from perception, end-effector mechanics, planning, or their interaction. Zhu et al. compared five robotic systems with different end-effectors in a bin-picking benchmark for robotic-assisted food handling in line production and found that end-effector architecture strongly affected pick-and-place and packing performance [112]. Related studies have compared end-effector performance across different food items [120] and used sensor-equipped food replicas to assess the success or failure of robotic pick-and-place operations [122]. Relevant measures include grasp and placement success, product damage, cycle time, failure recovery, and robustness to changes in product arrangement.

Technical task success must also translate into useful production performance. Busia et al. proposed an AI-driven architecture integrating a collaborative robot, camera, and image segmentation for handling food-packaging cardboard and identifying contaminated regions [123]. Accorsi et al. evaluated the technical, ergonomic, and economic feasibility of introducing a collaborative robot at the end of a food-catering production line. Based on a 30-iteration Monte Carlo analysis of the case-study assumptions, the authors estimated a 70% probability of achieving a payback period shorter than two years [124]. The estimated payback was sensitive to the production-lot size and the number of lots processed per day.

Coordination becomes another constraint when several high-speed robots share the same workspace or downstream process. Shared and non-deterministic material flows require system-level allocation, collision avoidance, and real-time coordination rather than independent optimisation of individual manipulators [125]. Line throughput then depends on synchronisation, task allocation, downstream capacity, and robot speed together. Faster manipulators provide limited benefit when shared access, product assignment, or downstream handling becomes the dominant bottleneck.

Across sorting and packaging applications, the principal challenge is converting perception into reliable physical action. High detection or segmentation accuracy improves target identification, but task performance also depends on localisation, grasp compatibility, product mechanics, contact stability, motion timing, and placement. Three-dimensional sensing can reduce spatial ambiguity, multimodal feedback can resolve states that change after contact, synthetic data can broaden perceptual coverage, and compliant mechanics can absorb part of the geometric mismatch. Their value should be judged by whether they improve repeatable recognition, grasping, transfer, placement, product integrity, cycle performance, and robustness across changing products.

This task-chain view also provides a practical basis for system improvement. Reliable detection followed by poor physical acquisition points to contact geometry, accessibility, or force regulation rather than another increase in detector accuracy. Stable grasping followed by placement failure at higher conveyor speeds shifts attention to timing, trajectory generation, or downstream coordination. Reporting stage-specific success rates and failure causes makes comparisons among systems more informative and reduces the risk that strong upstream metrics mask weak downstream operations [118,120,122]. This diagnosis is particularly important for foods because small changes in deformation, adhesion, or presentation can shift the dominant failure from one task stage to another.

### 4.3. Mobile Handling and Intralogistics

Robotic intralogistics extends automation from fixed workstations to the movement of products, packaging materials, and other items across production and logistics spaces [71]. The task involves more than transport between two locations: identification, loading, movement, destination assignment, and unloading must remain coordinated with the surrounding production process [126]. The required level of autonomy depends largely on variation in product presentation, route, destination, and nearby activity [71,81].

In highly structured environments, material transfer can be integrated directly into the production line without mobile autonomy. Qian et al. developed a machine-vision-guided system for positioning and tracking desiccant or oxygen-absorber packets before insertion into food cans [127]. The constrained task reduced dependence on manual placement and relied mainly on synchronisation among visual positioning, robotic transfer, and the moving production process. Fixed transfer remains effective when source and destination locations are stable and material routes are well defined.

Mobility becomes useful when products or materials must be handled across changing locations. Zia Ur Rahman et al. developed a vision-based mobile manipulator for handling and transporting supermarket products, using barcode recognition to identify product sections and SIFT descriptors for object detection [128]. Sakai et al. used a mobile dual-arm robot with universal vacuum grippers to stock and dispose of packaged items in a convenience-store environment [129]. The system selected single- or dual-arm manipulation according to the placement task, allowed re-grasping when needed, and achieved reported success rates of 100% for stocking and 80% for disposal. Both studies provide transferable rather than direct food-manufacturing evidence. They show that perception, navigation, grasp adaptation, and placement planning must remain coordinated as the robot moves between source and destination locations. Manufacturing deployment would impose additional demands from shorter cycle times, denser traffic, repeated operation, and stricter hygienic conditions.

Mobile transport and mobile manipulation impose different system requirements. A transport platform primarily moves material between stations; a mobile manipulator must also acquire and release products at each interface. This couples base localisation with end-effector positioning and makes docking, loading, and unloading part of the manipulation problem [126,130]. Reaching the correct station provides limited benefit if the robot cannot align reliably with the product, conveyor, rack, or destination.

Autonomous mobile robots become more valuable as routes, destinations, and production schedules vary because transport can be reassigned without permanently fixing the material-flow layout [71]. This flexibility introduces localisation, obstacle avoidance, traffic management, scheduling, and coordination requirements. A robot that waits for unavailable material, interferes with another process, or fails during loading can shift rather than remove the production bottleneck. Mobility should therefore be assessed across the complete logistics sequence, including loading and unloading success, delivery timing, waiting time, route conflicts, repeated-cycle reliability, and compatibility with production scheduling [81,126].

Evidence from food-manufacturing facilities further illustrates that the value of mobile automation depends on integration with established material flows. Stefanini and Vignali assessed the replacement of conventional forklift transport with an AGV-based alternative in an Italian food company, comparing environmental impacts and lifecycle costs [131]. This food-industry case complements the retail and general-robotics examples by providing direct evidence on the industrial context of internal transport. Its economic and environmental findings should not be interpreted as validation of autonomous product grasping or the long-term reliability of mobile manipulation.

Food-production environments add hygienic constraints to these interfaces. Surface protection, washdown compatibility, food-grade lubricants, and hygienic zoning may be required according to the level of product exposure [132,133]. Transport of sealed packages through dry areas imposes different requirements from operation near exposed foods or within washdown zones.

At production scale, the dominant limitations often occur where transport interfaces with processing rather than during navigation itself. Docking tolerance, conveyor or rack alignment, loading height, hand-off timing, queue formation, and receiving-station availability can determine whether mobile automation improves material flow [81,126]. These interfaces also determine where product exposure and hygienic zoning become important. Robotic intralogistics should therefore be evaluated through material-flow continuity rather than navigation accuracy or transport speed alone. Fixed transfer remains appropriate when routes and destinations are predictable; mobile platforms become more valuable as spatial and operational variability increases. Additional mobility is justified when its flexibility outweighs the associated demands for localisation, docking, coordination, scheduling, and hygiene. Representative integrated robotic systems are compared in Table 4, with emphasis on complete task performance and the principal evidence boundary of each study.

## 5. Opportunities and Challenges for Adaptive Food Robotics

### 5.1. Task-Level Reliability and Industrial Constraints

A central challenge in food robotics is maintaining reliable task performance under variable products and production conditions. Compliant grippers, three-dimensional and multimodal sensing, high-speed vision, adaptive control, and learning-based manipulation have expanded the range of foods that robots can detect and handle [1,2]. Industrial translation remains difficult because foods are not standardised mechanical objects. Postharvest condition and product formulation can alter maturity, surface characteristics, texture, and mechanical properties [138,139,140], which may in turn affect contact behaviour and damage susceptibility during robotic handling. The practical question is whether the complete robotic system maintains performance when these sources of variation occur together.

Manufacturing conditions expose the interaction of limitations that are often examined separately. Product variation changes contact requirements [15,24], while increasing conveyor speed can compromise online visual detection performance [56]. Even when recognition is sufficiently accurate, physical grasping may remain a limiting stage, as demonstrated in the catering-package assembly study [118]. Such failures may interrupt production and increase intervention requirements, making sustained line performance a central industrial concern.

Evaluation should connect component performance with the complete manipulation sequence. Detection accuracy, grasp success, force error, trajectory deviation, and cycle time remain useful, but each captures only part of production performance. Task-level assessment should include recognition and localisation, physical grasping, transfer, placement or release, product damage, cycle time, failure recovery, and robustness to changes in geometry or arrangement [30,120,122]. Repeated operation is equally important because sensor drift, material fatigue, residue accumulation, calibration changes, and altered contact behaviour may not appear in short laboratory trials. A system that performs well in tens of demonstrations may still require frequent intervention across product batches or sanitation cycles.

Failure recovery deserves explicit attention because the production consequence of an error depends on what happens after failure. A missed or partial grasp may be recovered through re-localisation and a second attempt, but a dropped, damaged, or incorrectly placed product may require rejection, line intervention, or cleaning before production resumes. Incomplete release, jams, temporary sensor loss, and docking errors create similar demands. First-attempt success alone cannot distinguish systems that recover autonomously from those that require operator intervention outside nominal conditions. Recovery rate, recovery time, and downstream effects should complement conventional success and cycle-time metrics [136].

Hygienic design is part of mechanical reliability rather than a separate compliance issue [6]. Food-contact grippers and nearby components may be exposed to residues, moisture, detergents, and repeated sanitation. Features introduced to improve manipulation can create additional hygienic burdens: surface textures may retain residues, variable-stiffness systems can require additional sealing, and embedded tactile sensors introduce interfaces that must remain protected during cleaning. Repeated cleaning and sterilisation have been shown to alter medical-grade silicone, although their implications for food-contact grippers require further validation [141]. Hygiene should be incorporated during system design rather than added after mechanical optimisation [6,133]. The force-controlled hygienic gripper reported by Pettersson et al. remains instructive because manipulation flexibility and decontamination were evaluated within the same system [7].

Industrial feasibility depends on sustained system performance rather than technical task success alone. Deployment costs include the robot, custom end effectors, sensing hardware, programming, calibration, integration, cleaning, maintenance, changeover, and production downtime. A technically advanced system may remain unattractive if product changes or sanitation require frequent specialist intervention. Modular end effectors can simplify changes in contact strategy [16], while distributed robotic architectures may facilitate data sharing and adaptation across production settings, although reductions in reconfiguration effort remain to be demonstrated [142]. These strategies are valuable only when they reduce changeover and maintenance burdens without compromising sanitation or task performance.

Industrial reliability should ultimately be judged over commissioning, production, changeover, sanitation, and maintenance. Lengthy recalibration after cleaning, frequent tool replacement, sensor fouling, material ageing, or specialist reprogramming can reduce effective production time even when cycle-level metrics are strong [16,141]. The relevant outcome is sustained output of acceptable products with predictable intervention requirements, not peak performance under controlled conditions. This system-level perspective also gives economic assessments, such as that of Accorsi et al., greater relevance because useful throughput is determined by the complete cell across its operating schedule rather than nominal robot speed alone [1,124].

### 5.2. Generalisation and Transferable Adaptive Robotic Intelligence

A further challenge in food robotics is reducing dependence on predefined products, workspaces, and manipulation sequences. Many systems still require new datasets, retraining, tool changes, recalibration, or manual programming when product categories or operating conditions change [16,142]. Strong performance on a fixed task does not necessarily indicate flexibility across batches, products, or production requirements. Generalisation requires useful performance to be retained when visual appearance, spatial arrangement, or physical interaction differs from development conditions. This is particularly demanding in food handling because visually similar products can differ in stiffness, friction, moisture, maturity, and damage tolerance enough to change grasp outcome [15].

Embodied and multimodal AI architectures offer potential routes to more flexible task representation by combining visual information, language, planning, and robot state [143]. Most have been developed in general robotics and should be treated as prospective strategies rather than evidence of industrial food-robot performance. OK-Robot, for example, combines vision–language-based detection with navigation and grasping for zero-shot pick-and-drop tasks. Across 171 trials in 10 real homes, it achieved 58.5% average success, increasing to 82.4% under more favourable query, scene, and object conditions [144]. This result illustrates both the potential and current sensitivity of open-vocabulary manipulation. Food handling adds further constraints from deformation, wet surfaces, allowable contact forces, and sanitation [15].

Language-conditioned planning extends adaptability from object recognition to task interpretation. VoxPoser uses language and vision–language models to generate instruction-conditioned three-dimensional value maps for closed-loop planning [145], and PaLM-E integrates language, vision, and robot state within an embodied reasoning architecture [146]. Such approaches may reduce the need for separate policies for every product or instruction. Their relevance to food manufacturing depends on whether high-level plans can also respect deformation limits, contact location, gripping force, tool compatibility, and cycle time. Large model size and computational demand make predictable latency an important deployment constraint.

These developments expose a distinction between semantic and physical adaptability. A robot may recognise an unfamiliar food, interpret a new instruction, and generate a plausible plan yet still fail because the grasp damages the product, the surface slips, or the action exceeds the available cycle time. Food-specific intelligence must incorporate product mechanics and contact constraints alongside semantic and visual information [16,117]. Generalisation should be tested not only across unfamiliar images or labels but also across changes in stiffness, friction, deformability, adhesion, and damage tolerance. Cross-batch and cross-condition validation is especially important because visual and mechanical shifts may occur independently.

The practical test of generalisation is whether the complete robot retains acceptable performance after relevant conditions change [120]. Food-sorting systems should be evaluated across batches, illumination conditions, product size, maturity, surface moisture, and presentation density, with both perception and manipulation outcomes recorded. Direct-handling systems should challenge the same grasp policy with realistic changes in contact mechanics rather than only new object labels [30]. Such tests can reveal whether adaptability comes primarily from the learned model, mechanical compliance, post-contact feedback, or their combination [16,117].

Adaptive intelligence also extends to navigation and computational deployment. Dual Graph Navigation combines external knowledge with an internally updated representation for visual target navigation [147], a principle that may apply when material locations or transport destinations change. FogROS2 separates computationally intensive planning from local robot execution and reduced grasp-planning time from 14 s to 1.2 s, with some motion-planning tasks accelerated by up to 45-fold through remote execution [148]. Remote computation remains dependent on communication latency and network reliability, so force regulation, collision avoidance, and other time-critical functions may still require local execution.

Adaptive intelligence is most valuable when it reduces product-specific programming without weakening physical reliability or real-time performance. Open-vocabulary perception, multimodal reasoning, knowledge-guided planning, and distributed computation can broaden task flexibility, but they do not replace suitable grippers, calibrated sensing, contact feedback, or hygienic hardware. Industrial value depends on whether broader task capability is retained across food batches and operating conditions with predictable latency, recoverable behaviour, and safe physical interaction [120,142]. Generalisation in food robotics should be demonstrated through manipulation outcomes, not only recognition accuracy or instruction understanding.

### 5.3. Uncertainty-Matched System Design

Across the technologies and applications reviewed above, greater robotic complexity is valuable only when it addresses a corresponding limitation of the target food process. Uniform packaged products following predictable trajectories may not require highly compliant grippers, multimodal sensing, or highly adaptive control. Fragile, deformable, slippery, randomly arranged, or frequently changing products can justify richer mechanical adaptation, feedback, and planning [2,15,16]. This relationship underpins an uncertainty-matched system design perspective in which robotic capability is selected according to process variability and dominant failure modes rather than technological sophistication alone. The perspective is intended as a practical logic for task matching, not as a universal theoretical model of robotic uncertainty.

Variability and uncertainty should be distinguished in this context. Variability describes physical differences among products or operating states, including changes in geometry, stiffness, surface condition, quality, position, or motion. Uncertainty is used here operationally to describe task-relevant variation that cannot be observed, predicted, or accommodated sufficiently by the available robotic capabilities, rather than uncertainty in a strictly probabilistic sense. Geometric variation may be absorbed through passive compliance, estimated through three-dimensional sensing, or require both when grasp accessibility changes substantially. Variation in stiffness, friction, or deformation may be managed through contact design over a narrow operating range, with force or tactile feedback becoming more useful when acceptable grasp conditions cannot be defined reliably before contact [2,16].

The same logic applies to sensing, platforms, and control, whose relevance depends on whether they resolve task-specific information, access, or coordination limitations [2,31,71,79,82]. Additional capability is valuable only when it changes a downstream decision or reduces a task-relevant failure; otherwise, it can increase calibration, computation, latency, maintenance, sanitation, and integration burdens without improving the outcome.

In practice, uncertainty-matched design begins with the expected operating range and the failures that matter within it. Table 5 links representative sources of product and process uncertainty to likely failure modes, task-relevant robotic capabilities, candidate technical solutions, and suggested validation endpoints. These relationships are task-dependent: geometric variability, for example, may be reduced through product singulation or accommodated through three-dimensional perception and compliant grasping [2,112]. The appropriate response depends on which approach improves task performance under the intended operating conditions.

Validation should follow the same logic. Systems designed for narrow, predictable tasks should demonstrate reliability across their defined operating range rather than be judged by capabilities the task does not require. The validation endpoints proposed in Table 5 should therefore be related to the failures that a technical response is intended to reduce. A reduction in force-control error, for example, does not by itself establish lower product damage [31], just as improved detection accuracy does not necessarily translate into successful grasping or placement [112,118]. Industrial claims should also include repeated cycles, representative failure recovery, sanitation exposure, and changeover conditions, which can expose limitations hidden in short trials. Validation should be matched to both the claimed adaptability and intended production context.

Some uncertainty is better removed at the process level than compensated for robotically. Singulation, fixturing, controlled illumination, conveyor pacing, or more consistent presentation can reduce variation before it reaches the robot [2]. Environmental structuring is not a lesser form of automation when it controls failure modes more reliably and economically than additional sensing, mechanics, or computation. Two production lines exposed to similar product variability may justify different solutions: one may constrain upstream variation and use a relatively simple robot, and another may accept greater variability and compensate through richer sensing, compliance, and feedback. Neither is intrinsically more advanced. The better design is the one that maintains product integrity, task completion, useful throughput, hygienic compatibility, and predictable operation with acceptable intervention and lifecycle cost [126].

An uncertainty-matched perspective links food properties, production conditions, robotic capabilities, process structuring, and validation within a single design logic. Claims of broader adaptability should be supported by broader operating conditions; deliberately constrained systems should be judged by the reliability and efficiency achieved within their intended range of use [82,122]. This matching provides a practical route for translating advances in grippers, perception, control, and adaptive intelligence into food-robotic systems whose complexity is justified by the process they are designed to serve.

## 6. Conclusions

Robotic sorting and handling are expanding from structured operations to foods and packages with greater variation in geometry, mechanical properties, surface condition, quality attributes, and spatial arrangement. Advances in compliant gripping, perception, robotic platforms, and closed-loop control have broadened the range of products that can be manipulated, yet the main challenge lies in coordinating these capabilities across complete tasks. Accurate recognition does not ensure a feasible grasp, successful contact does not guarantee stable transfer, and greater robot speed does not necessarily increase useful throughput. Food-robotic performance should be evaluated across recognition, localisation, grasping, transfer, placement or release, product integrity, and failure recovery rather than through isolated component metrics.

Taken together, the reviewed evidence motivates an uncertainty-matched system-design perspective. Uniform products following predictable trajectories may be handled effectively with relatively simple sensing, conventional end effectors, and established control strategies. Irregular, fragile, deformable, slippery, moving, or frequently changing products can justify greater mechanical adaptation, richer sensing, feedback, or planning. These capabilities should be selected according to the dominant failure modes and operating constraints of the target process rather than combined to maximise technological sophistication.

Future progress depends on demonstrating that such adaptability can be sustained under realistic food-manufacturing conditions. Cleanability, food-contact suitability, repeated sanitation, cycle performance, failure recovery, product changeover, maintenance, and economic feasibility should be evaluated alongside perception and manipulation accuracy. More general robotic intelligence may reduce dependence on product-specific programming, but reliable deployment still requires compatibility with food mechanics, damage limits, hygienic operation, and predictable response times. Industrial adoption will depend on whether robotic systems can accommodate the intended range of product and process variability while maintaining reliable, hygienic, damage-limited, and economically viable operation.

## Figures and Tables

**Table 1:** Representative end-effector strategies for robotic food handling and their capability trade-offs.

End-Effector Strategy	Food/Product or Task	Adaptation/Actuation Principle	Representative Performance	Main Capability	Main Trade-Off/Limitation	Representative Study
Pneumatic compliant + force control	Fragile-fruit grasping for postharvest handling	Pneumatic soft gripper + matrix force sensing + double closed-loop control	Maximum steady-state force error: 0.07 N	Gentle contact with closed-loop force regulation	Pneumatic response and sensing/control complexity	[31]
Sensorised compliant/bionic	Fragile fruits and vegetables	Six-finger compliant hand + contact-force and bending sensing	Slip detection and grasp-force optimisation demonstrated	Post-contact adaptation and slip-aware grasping	Sensor integration, wiring and calibration requirements	[11]
Underactuated/flexure-based	Circular objects (radius 30–40 mm)/agro-industry gripping	Push–pull flexure with passive self-adaptation	Radius 30–40 mm; pull-out force 21.4 N at 100 N maximum actuation force	Passive geometric adaptation with high pull-out force	Limited active contact-state regulation	[32]
Active material/DEA	Proof-of-concept grasping; paper cup	Dielectric-elastomer actuation	40° bending; 37.2 mN at 24 kV	Compact actuation within compliant fingers	High voltage and low output force; no direct food validation	[22]
Variable stiffness	Objects varying in shape, hardness and mass	PneuNet + particle jamming	0.124 s response; stiffness +92%/+107%; 5000 cycles; payload 9.8× self-weight	Soft conformity combined with enhanced load support	Additional vacuum, sealing and switching complexity	[26]
Pneumatic containment-based soft gripper	High-speed handling of breadcrumb-coated oysters	Pneumatic containment-based soft gripper; geometry and stiffness tailored for high-acceleration containment	Maximum operational speed ≈90/80 oysters min^−1^ for ≈16/40 g oysters under favourable orientation; soft-finger durability > 1.6 × 10^6^ cycles	High-speed, large-area compliant containment of highly soft and damage-prone foods	Performance depends on gripper stiffness, product orientation and operating speed; task-specific geometry limits generality	[4]
Octopus-inspired distributed suction	Underwater tofu and raw egg-yolk manipulation	Hydrogel tentacles + switchable biomimetic suckers	Nondestructive grasping and release demonstrated	Gentle distributed adhesion for ultra-soft products	Underwater proof-of-concept; practical hygiene/durability unresolved	[28]
Tendril/wrapping	Strawberry, slippery meat, pudding, caviar and raw egg yolk	Angle-programmed kirigami/tendril-like wrapping	Demonstrated manipulation of diverse soft, fragile and slippery foods	Distributed contact with broad geometric adaptability	Application-level cycle performance, release reliability and cleanability remain unvalidated	[33]

**Table 2:** Representative sensing and perception approaches for robotic food sorting and handling.

Sensing/Perception Approach	Food/Task	Task-Relevant Information	Processing/Model	Representative Performance	Robotic Role and Main Limitation	Representative Study
RGB machine vision	Online sweetpotato grading on a roller conveyor	Surface defects, size, object identity and multi-view tracking	YOLOv8l + BoT-SORT; instance segmentation and multi-view grading	HOTA = 0.952; length/width estimation accuracy = 96.7%/90.3%; final four-class grading accuracy = 91.7%	Supports online grading, tracking, and downstream routing; performance remains sensitive to illumination, appearance variation, and inference conditions	[55]
Hyperspectral imaging	Apple bruise detection and quality sensing	Spectral-spatial information related to bruise, chlorophyll, sugar and intrinsic material properties	On-chip computational VIS–NIR hyperspectral imaging	HyperspecI-V2 sensor specifications: 400–1700 nm, 96 spectral channels, 1024 × 1024 pixels at 124 fps, with an average light throughput of 74.8%.	Provides high-speed spectral information unavailable from RGB; robotic sorting-line integration was not demonstrated, and reconstruction, calibration and illumination stability remain deployment considerations	[42]
NIR + Raman spectroscopy	Salmon freshness assessment	Intrinsic freshness represented by TVB-N	Low-level/mid-level spectral fusion + LSSVM	Best low-level fusion model: R^2^P = 0.910, RMSEP = 1.922 mg/100 g	Suitable for intrinsic-quality assessment; non-imaging spectroscopy provides no spatial localisation, and robotic/online sorting integration was not demonstrated	[51]
Binocular structured-light 3D sensing	Apple morphology measurement for grading	Deformity, maximum diameter, shape, volume and mass	Binocular structured light + multi-view point-cloud reconstruction	Depth error ≈ 0.5 mm; R^2^ = 0.9941, 0.9594, 0.8015, 0.9915, 0.9960 for deformity index, maximum diameter, shape index, volume and mass, respectively	Provides detailed geometry potentially useful for grading and grasp planning; multi-view rotary-table reconstruction increases acquisition and processing requirements	[53]
Active laser depth sensing	Piled Chesapeake blue crabs and white-button mushrooms	High-resolution surface depth under partial occlusion	Dual line-laser scanning + galvanometer-based active scanning	Reference-stage reconstruction: MSE = 0.3 mm, STD = 0.5 mm; dual-laser scanning reduced occlusion gaps in piled crab and mushroom depth maps	Useful for item separation and localisation in piled products; requires dedicated active scanning, calibration and reconstruction hardware	[54]
High-speed online vision	Foreign-material detection in fresh-cut cabbage and green onion	Moving-object detection under conveyor operation	CMOS camera + YOLOv7x + conveyor control	At 36.73 cm s^−1^: 97.92%/95.00% accuracy for cabbage/green onion; at 69.88 cm s^−1^: 87.88%/86.67%	Demonstrates the coupling between perception and conveyor speed; accuracy decreases at high speed because of motion blur and occlusion, while robotic contaminant removal remains to be integrated	[56]
RGB + LiDAR multimodal 3D perception	Dynamic non-destructive grasping of thin-skinned fruits	Classification, instance segmentation and 3D spatial structure	RGB feature extraction + LiDAR point cloud + multimodal depth-fusion CNN	Multimodal perception was integrated with grasp-configuration planning and forward-looking nondestructive control, outperforming comparison approaches across most evaluated grasping criteria	Integrates appearance and 3D spatial perception with grasp planning and control; evidence is from agricultural fruit manipulation rather than factory food-sorting conditions	[62]
Tactile + pneumatic-state sensing	Adaptive grasp-force estimation for fragile fruits	Contact state and product-specific required grasping force	Tactile + air-pressure inputs; CNN/LSTM/Transformer-based LAFNet	Inference time 1.20 ms; adaptive grasp-force estimation error 0.82 ± 0.71 N	Enables product-specific post-contact force adaptation; requires an additional pinching/pre-grasping sensing stage, while long-term food-contact durability and hygienic integration remain unvalidated	[69]

**Table 3:** Representative control and coordination approaches for robotic food sorting and handling.

Control/Coordination Level	Food/Task	Control Strategy	Representative Outcome	Task-Level Role	Main Limitation/Evidence Boundary	Representative Study
Trajectory tracking control	Parallel robot for food sorting	Sliding-mode control + fuzzy control + improved bat-algorithm optimisation	Sorting accuracy of 99.90%; average sorting time of 0.509 s	Maintains repeatable high-speed motion and improves disturbance rejection during sorting	Performance demonstrated for a specific 4-DOF parallel sorting platform; transfer to variable foods and integrated sensing must be separately validated	[75]
Time-optimal trajectory planning	UR5-based food-sorting robotic arm	3-5-3 polynomial interpolation + improved coati optimisation algorithm	Mean simulated trajectory execution time: 7.883 s; 14.58%, 21.20%, and 4.18% lower than those of three comparison methods, respectively	Reduces nonproductive robot motion while respecting joint angle, velocity and acceleration constraints	Simulation-based validation; physical food sorting, food-contact interaction, and production-line operation were not experimentally demonstrated	[83]
Closed-loop grasp-force control	Fragile-fruit grasping	Matrix force sensing + Kalman filtering + double PID loops with dead band	Maximum steady-state force error of 0.07 N	Adjusts interaction force after contact to limit damage while maintaining grasp stability	Pneumatic time delay limits transient response; matrix sensing and double-loop control increase integration complexity	[31]
Robust visual servoing	Cucumber harvesting under fruit/pedicel motion	Video stabilisation + FSBF + FPFH-based 6D pose estimation + robust visual servoing	Perception accuracy of 90.00%; approach accuracy of 82.22%; detection of 15–37 fps	Continuously updates manipulator approach to compensate for target motion and visual disturbance	Approach accuracy remains below perception accuracy; demonstrated in harvesting rather than postharvest manufacturing	[79]
Continuous locomotion–picking coordination	Mobile fruit-harvesting robot operating throughout an orchard	ORB-SLAM3-based localisation/multi-map fusion + dynamic visual picking + behavioural coordination	Continuous autonomous locomotion and picking were experimentally demonstrated under field conditions	Coordinates navigation, localisation and manipulation instead of optimising each module independently	Agricultural-field evidence; coordination logic is transferable, but not direct validation in food-manufacturing layouts	[80]
Robust arm control + dual-arm coordination	Dual-arm apple harvesting	Quintic trajectory generation + robust trajectory tracking + failure-aware dual-arm coordination using pressure feedback	Field success rates were 80.7% and 79.7%; mean picking cycle of 5.97 s; coordination reduced harvest time by 28% versus single-arm baseline	Links arm-level control with system-level resource and failure coordination	Orchard harvesting differs from factory sorting; canopy/branch interference remained a failure source, and movement through the orchard still required manual operation	[77]
Multi-robot task and path coordination	Heterogeneous warehouse robots	Integrated task assignment + conflict-free path planning on a state–time–space network; Lagrangian-relaxation heuristic	Proposed heuristic outperformed the baseline on simulated and industrial instances	Coordinates shared resources, routes and heterogeneous robots at the system level	Adjacent-domain evidence rather than food-specific validation; benefit depends on production-layout and traffic structure	[82]

**Table 4:** Representative integrated robotic systems for food sorting and handling, with task-level performance and evidence boundaries.

Food/Product and Task	Robot/Platform	End Effector/Sorting Mechanism	Perception and Intelligence	Integrated Task Performance	Main Limitation/Evidence Boundary	Representative Study
HMR pouch loading onto retort trays	Doosan H2017 articulated collaborative robot	30-pad vacuum gripping system	Tray/conveyor sensing; simulation-based trajectory and production-flow optimisation	Actual throughput increased from 3133 to 4110 packs h^−1^ (+31.2%) in Case A and from 648 to 723 packs h^−1^ (+11.6%) in Case B; workers reduced 3 → 1 and 2 → 1, respectively	Strong real-factory evidence, but task is highly structured with fixed product presentation and trajectories	[101] (D)
Bin-picking and pick-and-place of deformable poultry pieces	ABB IRB 1200 6-DoF manipulator	Festo adaptive Fin-Ray gripper fingers	Intel RealSense D435 RGB-D + Mask R-CNN instance segmentation + pose/grasp estimation and motion planning	Overall pick-and-place success: 49.4%; reported module-level failure rates were 21.8% for perception, 30.7% for gripping, and 11% for manipulation.	Clutter, deformability and sticky/slippery surfaces substantially reduced system reliability; gripping was a major failure source and validation remained at experimental scale	[134] (D)
Online sweetpotato quality grading and sorting	Roller-conveyor automated sorting prototype	Pneumatically actuated sorting mechanisms	YOLOv8l multi-view segmentation, tracking, size/defect grading	At 12 cm s^−1^, grading accuracy 93.8% and integrated sorting accuracy 92.7%; sorter alone achieved 96.9% accuracy and 92.5% repeatability	Performance decreased as conveyor speed increased; prototype requires further commercial-line validation	[135] (A)
Tomato detection/classification and robotic handling	UR3e collaborative robot	Robotiq 2F-85 actuator with Fin-Ray-effect soft fingers	Raspberry Pi 5 + PiCamera 3; TensorFlow Lite object detection	Of 640 manipulation attempts, 390 were safely successful, 80 succeeded near the kinematic limit, and 170 triggered collision/unreachable conditions; reliable workspace = 262 × 250 mm	Failures were dominated by robot reach/kinematics rather than detection; benchtop system rather than continuous production line	[136] (A)
Recognition and sorting of reflective/transparent food packages	Vision-guided robotic arm	Conventional robotic grasping end effector	Mask R-CNN + structured-light 3D reconstruction + hand–eye coordinate transformation	Reported prediction examples from validation-set images achieved a 100% correct rate with no false checks; the final mixed-package demonstration (13 packages) achieved a 100% correct grasping rate.	Small dataset (300 images) and controlled laboratory tests; broader batch/product generalisation remains unverified	[113] (D)
Oyster size classification and tray placement for freezing	Delta/parallel robot + conveyor	Soft, drainage-textured gripper	Vision darkroom + photo sensor; image-moment-based size and grasp-point estimation	Classification and grasping success were both 99% over 100 oysters; process simulation assumed a 0.4 s robot cycle and estimated 1472 filled trays per 8 h for one system	Tests avoided oyster overlap; moisture-induced slip and escape of small oysters remained failure modes; the 8 h productivity estimate was simulation-based	[137] (D)
Grocery pick-and-pack with variable rigid and deformable products	MiR100 + UR16e mobile manipulator	Robotiq 2F-85 gripper	Eye-in-hand ZED 2i; YOLOv11-Nano + FastSAM-s + PCA grasp-pose estimation on Jetson Orin Nano	Level-3 benchmark: 92% overall success (23/25); average execution time ≈ 7.5 s; deformable sweet bag only 60% success	Integrated system-level evidence, but Level-3 testing used only five trials per item and was conducted in grocery benchmark/competition settings rather than an industrial food-processing line; deformable items remained challenging	[102] (T)
Dual-arm apple picking and postharvest transfer (adjacent agricultural evidence)	Two 4-DOF arms + movable platform	Centralised vacuum harvesting system	ToF camera; foundation-model detector + segmentation + depth estimation; pressure-feedback coordination	Two commercial orchards: success 80.7%/79.7%; mean cycle 5.97 s apple^−1^; dual-arm coordination reduced harvesting time by 28% versus single-arm baseline	Field-scale integrated evidence is strong, but orchard harvesting is transferable evidence rather than direct food-manufacturing validation	[77] (A)

Note: D, direct food-manufacturing task evidence; A, adjacent agricultural/postharvest evidence; T, transferable general-robotics evidence. Categories indicate application context rather than the level of industrial validation.

**Table 5:** Evidence-informed uncertainty-matched design matrix for robotic food sorting and handling.

Source of Product/Process Uncertainty	Potential Task Failure Modes	Task-Relevant Robotic Capabilities	Candidate Technical Responses	Suggested Validation Endpoints
Geometric and presentation variability: irregular shapes, size variation, clutter and overlap	Localisation or grasp-planning errors; inaccessible grasps; failed acquisition or placement	Spatial estimation, grasp planning and adaptable contact	Controlled product presentation or singulation; 3D vision; compliant grippers [62,112,134]	Pick-and-place success; stage-specific failure rates; product damage; cycle time under varied arrangements
Mechanical and surface variability: stiffness, moisture, friction and adhesion	Slip, excessive contact loading, unstable transfer and product damage	Contact adaptation and post-contact force regulation	Compliant contact interfaces; tactile/slip sensing; closed-loop force control [11,31,88]	Grasp and transfer success; slip incidence; product damage; force-control error under varied product states
Intrinsic-quality variability: compositional differences and defects not reliably visible in RGB images	Incorrect grading or rejection decisions	Non-destructive assessment of task-relevant quality attributes	Multispectral or hyperspectral imaging; NIR/Raman spectroscopy; application-specific calibration [40,42,51]	Agreement with reference quality measurements; robustness across batches; decision latency; routing accuracy if integrated with a sorting system
Dynamic production conditions: changing conveyor speed, target motion and illumination	Motion blur, missed detections, inaccurate target localisation and unsuccessful sorting	Timely detection, tracking and coordinated actuation	High-speed vision; object tracking; visual feedback; conveyor synchronisation [56,79,135]	Detection and integrated sorting accuracy across operating speeds; missed detections or sorting actions; useful throughput
Material-flow and layout variability: changing routes, destinations and loading/unloading requirements	Transfer-interface failures, delays, congestion and interrupted material flow	Reliable material transfer, coordination and scheduling	Fixed transfer where appropriate; AGVs/AMRs; loading/unloading interface design and coordinated scheduling [71,126,131]	Delivery and transfer success; waiting time; material-flow throughput; operating cost and environmental impact where relevant

Note: This matrix synthesises evidence from the reviewed literature rather than a universally validated design model. The suggested validation metrics are application-dependent and were not necessarily reported in the cited studies. Hygienic compatibility and operational reliability remain cross-cutting considerations.

## Data Availability

No new data were created or analyzed in this study. Data sharing is not applicable to this article.

## Supplementary Materials

The following supporting information can be downloaded at https://www.mdpi.com/article/10.3390/foods15213991/s1, Table S1: Web of Science Core Collection search strategies used for supplementary literature retrieval.
