# Supplementary material for: Robotic Sorting and Handling in Food Manufacturing: Enabling Technologies, System Integration, and Industrial Challenges
Source: Foods. 2026 Nov 6;15(21):3991. doi: 10.3390/foods15213991 (PMC13763406; doi:10.3390/foods15213991)
Supplement: Supplementary file 1 [file foods-15-03991-s001.zip › foods-4620091-supplementary.pdf]

Supplementary Table S1. Web of Science Core Collection search strategies used for supplementary literature retrieval.

Search group	Research focus	Full WoS topic-search expression	Records (n)
**S1**	Robotic applications in food manufacturing	TS=(("food processing" OR "food manufacturing" OR "food production" OR "food sorting" OR "food packaging" OR "food handling") AND (robot* OR "robotic manipulation" OR "robotic picking" OR "pick-and-place"))	442
**S2**	Robotic grippers and end effectors for food handling	TS=(("robotic gripper*" OR "soft gripper*" OR "robotic end effector*" OR "underactuated gripper*") AND ("food handling" OR "food processing" OR "food manufacturing" OR "food packaging" OR "food sorting"))	47
**S3**	Perception, vision, and automated food sorting	TS=(("food sorting" OR "food grading" OR "food quality inspection" OR "food packaging") AND ("machine vision" OR "computer vision" OR hyperspectral OR "RGB-D" OR "3D vision" OR "object detection" OR "instance segmentation" OR tracking) AND (robot* OR conveyor* OR "automated sorting"))	23
**S4**	System integration, task performance, and reliability	TS=(("food manufacturing" OR "food processing" OR "food packaging" OR "food handling") AND (robot* OR cobot* OR "delta robot*") AND (throughput OR "cycle time" OR "grasp success" OR reliability OR hygien* OR "production line" OR "product damage"))	41
**S5**	Mobile robotics and intralogistics	TS=(("food manufacturing" OR "food processing" OR "food warehouse*" OR "food logistics" OR "food distribution") AND ("autonomous mobile robot*" OR "mobile manipulat*" OR "automated guided vehicle*" OR intralogistic* OR AGV))	7
**S6**	Agricultural and postharvest robotic applications	TS=(("agricultural harvesting" OR "robotic harvesting" OR "postharvest handling" OR "fruit picking" OR "grocery picking") AND (robot* OR "robotic grasp*" OR "robotic handling") AND (grasp* OR gripp* OR "visual servo*" OR "motion planning" OR "tactile sens*"))	269
**Total**	**Before deduplication**	**Six separately exported search sets**	**829**

Note. TS denotes the Web of Science Topic search field. Searches were conducted in the Web of Science Core Collection on 17 October 2026, with publication dates restricted to 1 January 2015–17 October 2026. The six search sets contained 829 records before deduplication and 732 unique candidate records after deduplication by DOI, Web of Science accession number, and publication title. The 732 records constitute a supplementary literature search pool, not the number of studies finally included in this narrative review.
